# Determinants of HIV infection at 18 months of age among HIV-exposed infants in the context of PMTCT interventions in southern Ethiopia

**DOI:** 10.3389/frph.2024.1452889

**Published:** 2024-11-11

**Authors:** Eskinder Israel, Ayalew Astatkie, Kefyalew Taye, Aliki Christou, Ephrem Lejore, Anteneh Asefa

**Affiliations:** ^1^ School of Public Health, College of Health Science and Medicine, Wolaita Sodo University, Sodo, Ethiopia; ^2^ School of Public Health, College of Medicine and Health Sciences, Hawassa University, Hawassa, Ethiopia; ^3^ Department of Pediatrics and Child Health, School of Medicine, College of Medicine and Health Sciences, Hawassa University, Hawassa, Ethiopia; ^4^ Department of Public Health, Institute of Tropical Medicine, Antwerp, Belgium

**Keywords:** HIV, HIV-exposed infants, HIV-positive women, MTCT, PMTCT, outcome, Ethiopia

## Abstract

**Introduction:**

Mother-to-child transmission (MTCT) of HIV accounts for over 90% of annual HIV infections among children under the age of 15. Despite the introduction of the Option B+ strategy in Ethiopia in 2013, the rate of MTCT of HIV at 18 months was 15% in 2020. This study aimed to identify determinants of HIV infection among HIV-exposed infants (HEIs) in the context of prevention of MTCT (PMTCT) care in southern Ethiopia.

**Methods:**

We conducted a retrospective facility-based study of 299 mother-baby pairs (HIV-infected women and their HEIs up to 18 months) enrolled in PMTCT care at three health facilities in Wolaita Sodo town, southern Ethiopia, from September 2015 to October 2021. Data was collected from medical charts and PMTCT registers. Logistic regression was used to identify determinants of HIV infection among HEIs.

**Results:**

At enrolment into PMTCT care, most women were already on ART (75.3%) and in clinical stage I (89.6%) according to the World Health Organization's algorithm. Overall, 13 (4.3%, 95% CI: 2.5%–7.4%) HEIs were HIV-infected; the level was higher among HEIs born at home (17.9% (95% CI: 7.6%–36.5%). Being born to HIV-positive women with late WHO clinical stage (III and IV) of HIV (AOR = 9.1, 95%CI: 2.4, 34.5), being born at home (AOR = 4.8, 95%CI: 1.1–20.7), being born to women newly diagnosed with HIV (AOR = 4.8, 95%CI: 1.3–17.4), and low infant adherence to cotrimoxazole prophylaxis (AOR = 5.4, 95%CI: 1.4–20.4) increased the odds of MTCT.

**Conclusion:**

HIV infection levels among breastfeeding HEIs in PMTCT care was <5%, meeting the WHO transmission rate targets. Strengthening PMTCT care to expand community-based PMTCT interventions such as improving women's and communities’ awareness of HIV, PMTCT and promoting male involvement would reduce reduce HIV infection among children to reach the 95–95–95 targets to end HIV in Ethiopia.

## Introduction

In 2019, more than 38 million people were estimated to be living with HIV globally, of which approximately 1.8 million were children aged under the age of 15 (1). Mother-to-child transmission (MTCT) of HIV during the periods of pregnancy, childbirth, or breastfeeding accounted for more than 90% of HIV infections among these children (2). In 2020, nearly 150,000 new HIV infections among children aged under 15 years were reported globally; three out of five of these infections occurred in sub-Saharan Africa (SSA) (3, 4). According to a recent HIV estimate in Ethiopia, a total of 41,788 HIV-infected children and 3,195 new HIV infections among children aged 0–14 years were reported in 2021 (5). While the risk of MTCT of HIV among exposed infants can range from 15%–45% in the absence of any PMTCT intervention, the risk can be greatly reduced (to less than 5% in breastfeeding populations) if effective and timely PMTCT interventions are implemented (6, 7). To reduce MTCT, the World Health Organization (WHO) launched the Option B+ strategy in 2013, which recommends that all HIV-positive pregnant women receive lifelong antiretroviral therapy (ART) (6). According to a 2016 WHO report, more than 76% of HIV-positive pregnant women worldwide were receiving ART to prevent the transmission of HIV to their infants (8). A recent report showed that integrating PMTCT services with other maternal health services reduces the risk of MTCT of HIV (9).

In 2013, the Ethiopian Federal Ministry of Health (FMOH) launched the Option B+ PMTCT strategy and adopted the UNAIDS 90-90-90 approach (global treatment target aimed to end the HIV epidemic by 2030) (10, 11). According to the 2016 Ethiopian Demographic and Health Survey (DHS), the prevalence of HIV in the general population was 0.9% (0.4% in the Southern Nations Nationalities and Peoples Region) (12). In addition to this, the percentage of HIV-positive pregnant women accessing ART in the country was 92% in 2020 (13). Despite the introduction of the Option B+ PMTCT strategy in Ethiopia, the rate of MTCT of HIV among HEIs was 15% in 2020 (8% at 6 weeks of age and 7% during breastfeeding) (8).

Previous studies in Ethiopia have reported varying levels of MTCT at 18 months of age, ranging from 15.7% in Diredawa in 2013 (prior to the introduction of the Option B+ PMTCT strategy) (14) to 27% in Bahir Dar Administration in 2018 (15). Although the implementation of Option B+ PMTCT in Ethiopia increased the percentage of HIV-positive pregnant women on antiretroviral treatment (ART) from 25% in 2010 to 73% in 2015, the prevalence of MTCT among breastfeeding HEIs increased from 24% in 2012 to 30% in 2015 (16). A WHO report also showed that although nevirapine administration to HEIs in Ethiopia increased from 20% in 2012 to 34% in 2015, the rate of MTCT increased to 18% over the same period (17). These estimates show that although great efforts have been made to reduce MTCT nationally, the HIV incidence among HEIs in Ethiopia remains high and disproportionately high in some regions of the country, indicating the need for urgent and multidimensional interventions. Despite growing evidence on the level of MTCT after enrolment in the Option B+ strategy from studies conducted in other regions of Ethiopia, there is limited evidence in southern Ethiopia. This study aims to contribute to the knowledge base by assessing the determinants of HIV infection among HEIs enrolled in PMTCT care in Southern Ethiopia.

## Methods

### Study design, setting and population

We conducted a retrospective analysis of facility-based data by reviewing medical records of mother-baby pairs who were enrolled in PMTCT care in three health facilities that provide PMTCT service in Wolaita Sodo town, southern Ethiopia. Wolaita Sodo is the political and administrative center of the newly established Southern Ethiopia Regional State and is located 380 km south of Addis Ababa, the capital of Ethiopia. At the time of the study, there were two hospitals (one public and one private), three public health centers, and thirty private clinics. Most of the private clinics provide clinically focused outpatient services while the public and private hospitals provide specialized inpatient and outpatient services. The private hospital is highly regarded in the community for the quality of its services.

Between September 2015 and October 2021, 326 mother-baby pairs (HIV-infected women and their HEIs) were enrolled in PMTCT care in the health facilities. Of these, we excluded 27 mother-baby pairs who had at least one of the following events: were lost to follow-up, transferred out, or lacked data on HIV confirmatory test for the HEI at 18 months of age or six weeks after cessation of breastfeeding for babies between 12 and 17 months of age. Our analysis included 299 mother-baby pairs (HIV-infected women and their newborns) enrolled in PMTCT care in all three-health facilities providing PMTCT services.

### Variables

The outcome of interest in this study was HIV serostatus of HEIs. This was based on an HIV antibody test of HEIs done at 18 months of age and above or six weeks after cessation of breastfeeding for those infants between 12 and 17 months of age (18) or an HIV test result identified with HIV Deoxyribonucleic acid polymerase chain reaction (DNA/PCR) test done at 6 weeks of infants’ age or above.

Independent variables included socio-demographic and reproductive characteristics (maternal age at enrolment, maternal age at PMTCT registration, place of residence, educational level, marital status, and parity); PMTCT care related characteristics obtained from maternal health records (women's status at enrolment into PMTCT service, ARV (Antiretroviral) regimen, total months on ART, maternal CD4 count, maternal viral load, WHO clinical stage of HIV, syphilis test result, TB (Tuberculosis) status, breast conditions, maternal ART adherence, spouse HIV status, gestational age, antenatal care (ANC) follow up, mode of childbirth, place of childbirth); and PMTCT care related characteristics obtained from infant health records [infant birth weight, intake of ARV prophylaxis, intake of CPT (cotrimoxazole preventive therapy) prophylaxis, CPT adherence, infant feeding practices, duration of breastfeeding, DNA/PCR test done, DNA/PCR test results, HIV antibody test results].

In Ethiopia, the level of ART adherence is assessed by a multi -method tool (including a combination of pill counting and self-reporting) that is used in resource-limited settings (19, 20). If these two methods yield discordant results, a self-reporting method, based on missed doses out of 60 doses, is used because of its low recall bias characteristics (20, 21). Three or fewer, four to eight, and nine or more missed doses are classified as good, fair and poor adherence, respectively. Adherence to CPT was also assessed using the same classification method.

### Data collection tools and procedures

We reviewed medical charts and PMTCT registers in the three health facilities and a data extraction sheet, which was adapted from the National Integrated MNCH/PMTCT Register logbook, HIV care intake forms, HIV care follow up forms, HEI charts and ANC registers were used to extract data on the mother-baby pairs (Supplementary File S1). Data was collected by the first author and checked by the research team. To ensure the appropriateness, simplicity, clarity, understandability, and coherence of the data extraction sheet, a pre-test was conducted using 5% (*n* = 15) of the total sample size at a nearby health center (Tebela Primary Hospital) before the actual data collection. Randomly selected samples of extracted data were also reviewed to check for possible inconsistencies.

### Data analysis

Data was entered and cleaned using EpiData version 3.5.1 and then exported to SPSS version 25, for further analysis. Descriptive statistics such as proportions, and means with standard deviations were used to describe variables of interest of mother-baby pairs. We carried out a logistic regression analysis to identify variables associated with HIV serostatus of HEIs. We first conducted bivariate logistic regression analysis and then included independent variables associated with the outcome at *p* < 0.25 in the multivariable logistic regression model. Five variables were included in the multivariable analysis. These were WHO Clinical HIV staging, place of birth, duration of breastfeeding, women's ART status at enrolment into PMTCT service, and HE's adherence to CPT. The Hosmer and Lemeshow test was used to check the model fit. Finally, odds ratios with 95% confidence intervals (CIs) were calculated to measure the strength of association between the independent variables and HIV serostatus of HEIs.

## Results

### Socio-demographic and reproductive characteristics of the women

Out of 326 mother-baby pair (HIV-infected women and their newborns) charts and registers available for review, twenty-seven (*n* = 27) were excluded due to incompleteness. As a result, 299 mother-baby pair charts and registers were reviewed. The mean (±SD) age of the women at enrolment into HIV care and PMTCT was 26.7 (±4.4) and 30.7 (±5.2) years respectively. The majority (81.6%; *n* = 244/299) of the women were aged 20–34 years when enrolled in HIV care. Almost two-thirds (63.9%; *n* = 191/299) of the women lived in Wolaita Sodo town and more than eighty percent (83.6%; *n* = 250/299) were married. One-fourth (25.4%; *n* = 76/299) of the women had completed secondary level education or higher. Eight (2.7%; *n* = 8/299) women had at least five or more births including the index birth. The mean (±SD) parity of the women was 2.31 (±1.00) (Table 1).

**Table 1:** Socio-demographic and reproductive characteristics of women.

Characteristics	Categories (*n* = 299)	*N* (%)
Maternal age at enrolment into HIV care (in years)	<20	31 (10.4)
	20–34	244 (81.6)
	≥35	24 (8.0)
Maternal age at PMTCT registration (in years)	<20	5 (1.7)
	20–34	220 (73.6)
	≥35	74 (24.7)
Place of residence	In Wolaita Sodo	191 (63.9)
	Out of Wolaita Sodo	108 (36.1)
Educational level	Not able to read and write	75 (25.1)
	Able to read and write	16 (5.4)
	Primary	120 (40.1)
	Secondary and above	76 (25.4)
	Not Recorded	12 (4.0)
Marital status	Single	12 (4.0)
	Married	250 (83.6)
	Divorced	28 (9.4)
	Widow/Widower	8 (2.7)
	Not recorded	1 (0.3)
Parity	1	50 (16.7)
	2–4	241 (80.6)
	≥5	8 (2.7)

### PMTCT service use and clinical characteristics of women

Three-fourths (75.3%; *n* = 225/299) of women were already on ART when enrolled on PMTCT care (Table 2). Almost two-thirds (65.6, *n* = 196/299) of women had been on 1-e (TDF + 3TC + EFV) as their recent ARV regimen and 62.9% (*n* = 188/299) had been on ART for a total of 12 months or more. Baseline CD4 count was done during the index pregnancy for 78.9% (*n* = 236/299) of women and was ≥350 /mm^3^ in 64.4% (*n* = 152/236). Similarly, viral load was measured for 64.9% (*n* = 194/299) of women and was undetectable in approximately 90% (90.7%, *n* = 176/194). Nearly ninety percent (89.6%; *n* = 268/299) of women were in WHO clinical stage I at the time of enrolment into PMTCT care. The majority of women (83.3%; *n* = 249/299) tested negative for TB, and less than five percent (3.3%, *n* = 10/299) tested positive for syphilis test in the index pregnancy. Nearly 90% of women (88.3%; *n* = 264/299) women had a normal breast condition during the index pregnancy and more than fifteen percent (16.7%, *n* = 50/299) of the partners of the women were sero-discordant. More than eighty percent (81.6%; *n* = 244/299) of women had good ART adherence.

**Table 2:** PMTCT service use and clinical characteristics of the women during the index pregnancy and postpartum period.

Characteristics	Categories (*n* = 299)	*N* (%)
Wome's status at enrolment into PMTCT service	Newly diagnosed	74 (24.7)
	On ART	225 (75.3)
ARV regimen	TDF + 3TC + EFV (1e)	196 (65.6)
	TDF + 3TC + DTG (1j)	57 (19.1)
		234 (74.1)
	Others	46 (15.3)
Duration on ART (in months)	<12	111 (37.1)
	≥12	188 (62.9)
CD4 taken	Yes	236 (78.9)
	<350	84 (35.6)
	≥350	152 (64.4)
	No	63 (21.1)
Viral load measured	Yes	194 (64.9)
	<1,000	10 (55.5)
	≥1,000	8 (44.5)
	Yes, undetectable	176 (90.7)
	No	105 (35.1)
WHO clinical stage of HIV	I	268 (89.6)
	II	28 (9.4)
	III	2 (0.7)
	Not recorded	1 (0.3)
Syphilis test results	Reactive	10 (3.3)
	Non-reactive	175 (58.5)
	Not detected	4 (1.3)
	Non recorded	110 (36.8)
TB status of the woman	Positive	3 (1.0)
	Negative	249 (83.3)
	Not detected	3 (1.0)
	Not recorded	44 (14.7)
women's breast condition	Normal	264 (88.3)
	Cracked nipples	20 (6.7)
	Mastitis	9 (3.0)
	Not recorded	6 (2.0)
women's ART adherence	Good	244 (81.6)
	Fair	16 (5.4)
	Poor	36 (12.0)
	Not recorded	3 (1.0)
Spouse HIV status	Positive	158 (52.8)
	Negative	50 (16.7)
	Unknown	42 (14.0)
	Not recorded	49 (16.4)

1e = Tenofovir disoproxil fumarate + Lamivudine + Efavirennz; 1J = Tenofovir disoproxil fumarate + Lamivudine + Dolutegravir.

### Obstetric characteristics of women

The majority (82.9%; *n* = 248/299) of women had attended at least one ANC visit during their index pregnancy and almost one-fifth (20.1%; *n* = 52/248) had attended four or more ANC visits (Table 3). Gestational age was recorded for more than three-fourths (78.9%, *n* = 236/299) and was ≥36 weeks for less than ten percent (8.5%, *n* = 20/236) of women. More than ninety percent (91.0%, *n* = 272/299) of women gave birth in a health facility and over three-quarters (78.3%; *n* = 234/299) had a spontaneous vaginal birth while the rest had a caesarean birth.

**Table 3:** Obstetric characteristics of women during their index pregnancy.

Characteristics	Categories (*n* = 299)	*N* (%)
Antenatal care visits recorded	Yes	248 (82.9)
	1	49 (19.9)
	2–3	147 (59.2)
	≥4	52 (20.9)
	No	51 (17.1)
Gestational age recorded (in weeks)	Yes	236 (78.9)
	<12 weeks	32 (13.5)
	12–23 weeks	91 (38.5)
	24–35 weeks	93 (39.5)
	≥36 weeks	20 (8.5)
	No	63 (21.1)
Mode of childbirth	Spontaneous vaginal delivery	234 (78.3)
	Caesarean section	55 (18.4)
	Not recorded	10 (3.3)
Place of childbirth	Health facility	272 (91.0)
	Home	26 (8.7)
	Not recorded	1 (0.3)

### Newborn practices and PMTCT care for HEIs

More than two-thirds (71.2%, *n* = 213/299) of HEIs had a normal birth weight between 2,500–4,000 g (Table 4). All the HEIs had received ARV prophylaxis; of which the majority (80.9%, *n* = 242/299) received nevirapine (NVP) for the first six weeks. Almost all (99.7%; *n* = 298/299) HEIs received CPT after 6 weeks of age until discharge from PMTCT care. Regarding infant feeding practices, 94.3% (*n* = 282/299) of HEIs were exclusively breastfed for the first six months of age while 2.0% (*n* = 6/299) had mixed feeding after the first six months of life; and 49.8% (*n* = 149/299) of HEIs were on breastfeeding for 12–18 months. Of those HEIs who received CPT prophylaxis, 251 (83.9%, *n* = 251/298) had good adherence.

**Table 4:** Newborn characteristics and PMTCT interventions for HEIs.

Characteristics	Categories (*n* = 299)	*N* (%)
Infant birth weight (in grams)	<2,500	85 (28.4)
	2,500–4,000	213 (71.2)
	≥4,000	01 (0.3)
Infant received ARV prophylaxis	Yes	299 (100)
	NVP	242 (80.9)
	NVP + AZT	57 (19.1)
	No	0 (0)
Infant received CPT prophylaxis	Yes	298 (99.7)
	No	1 (0.3)
Adherence to cotrimoxazole prophylaxis	Good	251 (84.2)
	Fair	16 (5.4)
	Poor	31 (10.4)
Feeding practice in the first six months of life	Exclusive breastfeeding	282 (94.3)
	Exclusive replacement feeding	9 (3.0)
	Mixed feeding	6 (2.0)
	Not recorded	2 (0.7)
Duration of breastfeeding (in months)	<12	37 (12.4)
	12–18	149 (49.8)
	>18	105 (35.1)
	Not recorded	8 (2.7)

### PMTCT interventions and outcome

All HEIs had received both DNA/PCR and HIV antibody testing. DNA/PCR test at or before six weeks of age was done for 65.9% (*n* = 197/299) of HEIs. A second DNA/PCR test was also done for two HEIs (0.7%, *n* = 2/299) for further confirmation of the results. Nine (3.0%; *n* = 9/299) HEIs were found to be positive by DNA/PCR testing. The mean age at HIV diagnosis for these HEIs was 7 weeks. Overall, thirteen (4.3%) HEIs were found to be positive by HIV antibody test at ≥18 months of age or six weeks after cessation of breastfeeding (12–17 months of age).

### Determinants of HIV infection among HEIs

The multivariable logistic regression indicated that WHO clinical staging of HIV, place of birth, women's status at enrolment into PMTCT service, and adherence to CPT were significantly associated with HIV serostatus of HEIs (Table 5).

**Table 5:** Logistic regression of variables associated with HIV infection among HEIs.

Variables	Category	HIV-positive serostatus of HEIs		COR (95% CI)	AOR (95% CI)
		Overall *N*	Positive *n* (%)		
WHO Clinical Staging of HIV	I	268	7 (2.3)	1	1
	II & III	30	6 (2.0)	9.32 (2.89–29.96)*	9.09 (2.39–34.46)**
Place of childbirth	Health Facility	271	8 (2.7)	1	1
	Home	28	5 (1.7)	7.14 (2.16–23.63)*	4.82 (1.12–20.71)**
Duration of breastfeeding (in months)	≤12	40	6 (2.1)	1	1
	>12	251	7 (2.4)	0.16 (0.05–0.51)*	0.14 (0.08–1.38)
Woman's ART status on enrolment	On ART	225	6 (2.0)	1	1
	Newly diagnosed	74	7 (2.3)	3.81 (1.23–11.73)*	4.78 (1.31–17.35)**
Infant's adherence to CPT	Good	251	6 (2.0)	1	1
	Poor	47	7 (2.3)	7.14 (2.28–22.35)*	5.39 (1.42–20.41)**

* Variables statistically significant at *p* < 0.25, **Variables statistically significant at *p* < 0.05, AOR, adjusted odds ratio; COR, crude odds ratio.

Women in WHO clinical stages II and III of HIV had over nine-fold increased odds of having an HEI with a positive HIV serostatus compared to women who were in WHO clinical stage I (AOR = 9.09; 95% CI = 2.39, 34.46). Compared with infants born in health facilities, infants born at home had increased odds of positive HIV serostatus (AOR = 4.82; 95% CI = 1.12, 20.71). The odds of MTCT of HIV were about four times higher among women who were newly diagnosed at PMTCT enrolment compared to those who were already on ART before PMTCT enrolment (AOR = 4.78; 95% CI = 1.31, 17.35). Infants with poor adherence to CPT had a five-fold increased odds of MTCT as compared to their counterparts (AOR = 5.39; 95% CI = 1.42, 20.41).

## Discussion

In this study, we found a HIV infection rate of 4.3% among HEIs at 18 months of age. Late clinical HIV stage of women, home birth, recent HIV diagnosis of women, and low infant adherence to CPT prophylaxis were associated with an increased risk of HIV infection among HEIs.

The level of HIV infection in the present study is in line with the WHO recommendation for the breastfeeding population (less than 5%) (2, 7). The rate was high compared to the rates in Ethiopia—Adama (0.4%) in 2017 (22), Northern Tigray (2.1%) in 2016 (23), Dessie (3.8%) in 2017 (18), Mekelle City (1.3%) in 2018 (24)—and Nigeria (2.8%) in 2016 (25). The lower HIV infection rate in the Adama study could be due to extensive multi-stakeholder support for HIV care and support program, resulting in relatively higher quality of HIV care. The lower rate in Northern Tigray, Dessie and Mekelle City studies may be due to differences in sociodemographic characteristics; most HIV positive women had a higher level of education compared to those in our study, which might have resulted in good uptake and adherence to PMTCT care. The MTCT rate among mother-baby pairs in our study was lower when compared to studies conducted in Diredawa (eastern Ethiopia), Gondar (northwest Ethiopia) (26), Woliso (central Ethiopia) (27), East and West Gojjam (northern Ethiopia) (28), South Omo (southern Ethiopia) (29), southwest Ethiopia (30), Sidama (southern Ethiopia) (31), Uganda (32), Rwanda (33), Congo (34), and South Africa (35). This could be because of the low (0.4%) HIV prevalence in the region, Southern Nations Nationalities, and Peoples Region (9) and the wide time span (2015–2021) of this study. The use of a prospective study design in Kigali (Rwanda), Durban (South Africa), and Congo (Central Africa) may have contributed to the difference observed in these African countries (33–35). Additionally, the low rate of MTCT of HIV observed in our study could be due to the inclusion of mother-baby pairs who were enrolled in option B+ PMTCT in recent years following the continuous strengthening of the PMTCT program in Ethiopia (36).

In our study, women in WHO HIV clinical stages II and III were more likely to have had an HEI with a positive HIV serostatus compared to women who were in clinical stage I. This is consistent with existing evidence indicating that advanced HIV stage is associated with the likelihood of new HIV variants and disease progression, as well as opportunistic infections (OIs) leading to an increased risk of vertical transmission of HIV (37, 38). This suggests that multidimensional efforts are warranted to improve the coverage of HIV testing before or during early pregnancy to initiate ART and other supportive care to minimize the risk of MTCT of HIV. Additionally, strengthening the quality of care to reduce poor adherence to treatment and loss to follow up remains an affordable strategy to reduce the risk of MTCT.

Compared with infants born in health facilities, infants born at home had an increased odds of MTCT of HIV. In the event of home birth, immediate initiation of ARV prophylaxis and safe childbirth practices may not be practiced in addition to the lack of immediate postnatal care for HEIs, resulting in an increased risk of vertical transmission of HIV (22, 39). This finding is supported by several studies conducted in Mekelle (24), Bahir Dar (15), Dessie (18), Addis Ababa (40), South West Ethiopia (30), North West Ethiopia (41), Uganda (32) and Kisii (Kenya) (42). This highlights the need for interventions to scale up community-level PMTCT services to improve the uptake of antenatal, intranatal, and postnatal care services among HIV-infected women.

In our study, the odds of MTCT were about four times higher among women who were newly diagnosed at PMTCT enrolment than among those who were on ART at enrolment. This is in line with existing evidence indicating that women who were on ART before their index pregnancy are more likely to have viral suppression before their index pregnancy. In contrast, newly diagnosed HIV-infected women are more likely to have high viral loads and low CD4 counts which increase the risk of vertical transmission of HIV (8–10, 38). This suggests that pre-conception care and counselling on MTCT (including HIV testing, education on risk factors, HIV risk, ART, PMTCT, drug toxicity, and safe sexual practices) is a cost-effective strategy to achieve and maintain suppression of viral load in pregnant women and maintaining it during pregnancy, childbirth, and the postpartum period. Growing evidence also supports the use of Dolutegravir (DTG)-based first line regimens by HIV positive pregnant and lactating women since it is found to reduce the incidence of MTCT as compared with Efavirenz (EFV)-based regimens (43, 44). Additionally, women of reproductive age who are HIV positive need to be on contraception such as hormonal implants at least once to prevent unwanted pregnancy and to achieve triple elimination of mother to child transmission of HIV, syphilis and Hepatitis B (45, 46).

Our study found that infants with low adherence to CPT had more than a five-fold increased likelihood of MTCT of HIV compared with infants with high adherence, which is similar to findings from a study conducted in Oromia Regional State (47). Poor adherence to CPT could expose HEIs to severe bacterial infections, such as pneumonia, and diarrhea, which could lead to mucosal lacerations, facilitating viral entry into the bloodstream (9, 39). WHO recommends that HEIs receive CPT from six weeks of age until HIV infection is ruled out or the HEI stops breastfeeding to reduce the incidence of opportunistic infections (6, 46, 48). However, maintaining optimal adherence to CPT is challenging in Ethiopia and other resource-limited settings due to stock-outs, women's poor knowledge of the benefits of CPT, and lack of adequate counselling (49–51). This highlights the need for multi-stakeholder interventions to strengthen the supply chain, train healthcare providers and health extension workers (HEWs) on PMTCT to provide optimal counseling and improve community-based follow-up systems.

A key strength of our study is the use of a retrospective design to collect as much information as possible from multiple data sources to identify the determinants of MTCT of HIV among mother-baby pairs enrolled in PMTCT care over a six-year period. However, it has several limitations. First, because the study used routine data sources, some mother-baby pairs had incomplete data. Second, the confidence intervals for the odds ratios calculated in our study are wide because of the small sample size and the small number of HIV-infected HEIs and should be interpreted with caution. Third, some of the key variables, such as adherence to ART and CPT, were based on women's recall and honesty which may have introduced recall and desirability bias especially given the sensitive nature of the issue. Finally, we were unable to disaggregate the exact timing of HIV infection among HIV-infected HEIs as the frequency of HIV testing along the HEI care was not optimal. We suggest that future studies should take these limitations into account to improve the robustness of studies on similar topics.

## Conclusion

Factors associated with vertical transmission of HIV among mother-baby pairs enrolled in PMTCT care in this study can be significantly reduced by strengthening the existing PMTCT care packages in Wolaita Sodo town, southern Ethiopia, and in Ethiopia in general. Future prospective studies are needed to determine the precise timing of HIV acquisition among HEIs. Concurrently, strengthening and scaling up community-based PMTCT interventions improving women's and communities’ awareness of HIV, PMTCT and promoting male involvement would reduce HIV infection among children and contribute to the achievement of the 95-95-95 targets to end the HIV epidemic in the country.

## Data Availability

The original contributions presented in the study are included in the article/Supplementary Material, further inquiries can be directed to the corresponding author.
