# Supplementary material for: Determinants of HIV infection at 18 months of age among HIV-exposed infants in the context of PMTCT interventions in southern Ethiopia
Source: Front Reprod Health. 2024 Nov 11;6:1452889. doi: 10.3389/frph.2024.1452889 (PMC11586338; doi:10.3389/frph.2024.1452889)
Supplement: Supplementary file 1 [file Datasheet1.docx]

**A study to determine HIV infection among HEIs at 18 months of age**

**Questionnaire Code: ______ Hospital/Health Center: __________________**

**Department: ------------------- Date of Data collected------------------------------**

1. **Socio-Demographic and Reproductive characteristics of the mother**

| **S.No** | **Variable** | **Response** | **Skip** | **Remark** | **Data source** |
| --- | --- | --- | --- | --- | --- |
|  | Hospital medical record number | **---------------** |  |  | *PMTCT register or*  *ANC register* |
|  | Age at enrollment to HIV care | _____ (in years) |  |  | *HIV care follow-up form* |
|  | Age at registration for PMTCT service | _____ (in years) |  |  | *PMTCT register or*  *ANC register* |
|  | Marital Status | 1. Single  2. Married  3. Separated  4. Divorced  5.Widow/Widower | All Except 2, skip to 106 |  | *Intake form* |
|  | Spouse HIV status | 1. Negative 2. Positive 3. Unknown |  |  | *Intake form* |
|  | Residence | 1.Outside Wolaita Sodo  2. Inside Wolaita Sodo  3. Not recorded |  |  | *Intake form* |
|  | Educational level | 1. Not able to read and write  2. Able to read and write  3. Primary  4. Secondary and above  5. Not recorded |  |  | *Intake form* |
|  | Parity | ----------------------- |  |  | *ANC register or*  *Integrated women's chart* |

**2. Mother's characteristics and HIV related care status**

|  | Date HIV tested  [DD/MM/YYYY]  ***Initial test which confirmed HIV positivity*** | **______/______/_____** |  |  | *HIV care follow-up form* |
| --- | --- | --- | --- | --- | --- |
|  | Date of enrollment into HIV care [DD/MM/YYYY] | **______/______/_____** |  |  | *HIV care follow-up form* |
|  | Date of enrollment into PMTCT care -[DD/MM/YYYY] | **______/______/_____** |  |  | *HIV care follow-up form or PMTCT register or*  *ANC register* |
|  | Mother's status at enrollment into PMTCT service | 1. On ART 2. On Pre-ART 3. Newly diagnosed | If 2 or 3, skip to 207 |  | *PMTCT register or*  *ANC register* |
|  | ART regimen | Regimen code________ |  |  | *HIV care follow-up form or PMTCT register or*  *ANC register* |
|  | Total months on ART | _______months |  |  | *HIV care follow-up form* |
|  | Date ART started [DD/MM/YYYY]  ***Whether naive or already on ART*** | **______/______/_____** |  |  | *PMTCT register or*  *ANC register* |
|  | CD4 taken | 1. Yes 2. No 3. Not recorded |  |  | *PMTCT register* |
|  | Baseline CD4 count during pregnancy  *(if more than two, use the earliest one)* | ------------ |  |  | *HIV care follow-up form or PMTCT register* |
|  | Viral load taken | 1. Yes  2. No  3. Not recorded |  |  | *PMTCT register* |
|  | Viral load during enrollment into PMTCT care (anytime) or then after in PMTCT care (specify the date) | **__________**copies/ml  **______/______/_____** |  |  | *HIV care follow-up form or PMTCT register* |
|  | Clinical stage of HIV upon enrollment into PMTCT care | 1. Stage I 2. Stage II 3. Stage III 4. Stage IV |  |  | *HIV care follow-up form or PMTCT register* |
|  | Gestational age at enrollment into PMTCT care | ________ weeks |  |  | *ANC register or*  *Integrated women's chart or*  *PMTCT register* |
|  | Antenatal care visit | 1. Yes  2. No |  |  |  |
|  | Total number of antenatal care visits | ________ |  |  | *ANC register or*  *Integrated women's chart* |
|  | Mode of childbirth | 1. SVD 2. Cesarean section 3. Forceps/Vacuum assisted 4. Episiotomy 5. Not recorded |  |  | *Delivery register or*  *Integrated women's chart* |
|  | Place of delivery | 1. Health facility 2. Home |  |  | *PMTCT register* |
|  | Maternal breast condition | 1.Normal  2. Mastitis/Breast Abscess.  3. Not recorded |  |  | *Integrated women's chart or*  *HEI follow-up card* |
|  | Syphilis test result | 1. Reactive  2. Not reactive  3. Not detected |  |  | *PMTCT register* |
|  | TB status | 1. Positive  2. Negative  3. Not detected |  |  | *PMTCT register* |
|  | ART adherence | 1. Good  2. Fair  3. Poor  4. Not recorded |  |  | *Integrated women's chart or*  *HEI follow-up card* |

**4. Newborn's characteristics and related HEIs care status**

|  | Date of birth [DD/MM/YYYY] | **______/______/_____** |  |  | *Delivery register/HEI follow-up card or/HEI register* |
| --- | --- | --- | --- | --- | --- |
|  | Birth weight | ________(in grams) |  |  | *Integrated women's chart or*  *HEI follow-up card* |
|  | Date of enrollment to HEI care [DD/MM/YYYY] | **______/______/_____** |  |  | *PMTCT register or*  *HEI follow-up card or*  *HEI register* |
|  | Did the baby receive ARV prophylaxis | 1. Yes 2. No | If 2, skip to 307 |  |  |
|  | Date ARV prophylaxis started [DD/MM/YYYY] | **______/______/_____** |  |  | *PMTCT register or*  *HEI follow-up card or*  *HEI register* |
|  | Type of ARV prophylaxis infant taken | --------------- |  |  |  |
|  | Did the baby receive CPT prophylaxis? | 1. Yes 2. No | If 2, skip to 309 |  | *PMTCT register or*  *HEI follow-up card or*  *HEI register* |
|  | Adherence to CPT prophylaxis | 1. Good  2. Fair  3. Poor  4. Not recorded |  |  | *PMTCT register or*  *HEI follow-up card or*  *HEI register* |
|  | Infant feeding practice within the first six months of life | 1. EBF 2. ERF 3. Mixed feeding |  |  | *HEI follow-up card* |
|  | Duration of breastfeeding in months |  |  |  |  |
|  | Was DNA-PCR sample taken for the infant | 1. Yes 2. No | If 2, skip to 313 |  | *PMTCT register* |
|  | Date first DNA PCR sample taken [DD/MM/YYYY] | **______/______/_____** |  |  | *PMTCT register or*  *HEI follow-up card* |
|  | DNA PCR Result | 1. Positive 2. Negative 3. Not recorded |  |  | *PMTCT register or*  *HEI follow-up card* |
|  | Was HIV antibody test done after 12 months of age? | 1. Yes 2. No |  |  | *HEI follow-up card* |
|  | Date HIV antibody test done [DD/MM/YYYY | **______/______/_____** |  |  | *PMTCT register or*  *HEI follow-up card* |
|  | Antibody test Result after 12 months of age | 1. Positive 2. Negative 3. Not recorded |  |  | *PMTCT register or*  *HEI follow-up card* |
|  | Date of initial HIV diagnosis ***(positive or negative)*** [DD/MM/YYYY] | **______/______/_____** |  |  | *PMTCT register or*  *HEI follow-up card* |
